# Associations between Handgrip Strength with Adverse Cardiometabolic Health among Representative Korean Adults

**DOI:** 10.3390/ijerph17010269

**Published:** 2019-12-30

**Authors:** Keun-Ok An, Junghoon Kim

**Affiliations:** 1 College of Humanities and Social Sciences, Korea National University of Transportation, Chungju 27469, Korea; koan@ut.ac.kr; 2 Sports and Exercise Medicine Laboratory, Korea Maritime and Ocean University, Busan 49112, Korea

**Keywords:** muscle strength, handgrip strength, cardiometabolic health, Korean, KNHANES

## Abstract

Reduced muscle mass and strength are notable features of aging. Loss of muscle mass contributes to cardiometabolic health. Although many studies have focused on skeletal muscle mass, it remains unclear whether muscle strength is beneficial for cardiometabolic health. We aimed to investigate the association between handgrip strength and the risk of adverse cardiometabolic health in middle-aged and older adults in the Korean general population. The study participants included 7602 adults from the Korea National Health and Nutritional Examination Survey (KNHANES). The odds ratio for adverse cardiometabolic health significantly and linearly decreased according to the category of handgrip strength adjusted for potential confounding factors (*p*-Value < 0.05). In the stratified models by sex we also observed significant associations between handgrip strength and cardiometabolic health in men (*p*-Value < 0.001), but not in women. Our findings suggest that maintaining higher handgrip strength may contribute to improved cardiometabolic health.

## 1. Introduction

Changes in body composition with age, including reduction of skeletal muscle mass and muscle strength, are important characteristics of aging. After the age of 40 years, the amount of skeletal muscle decreases by approximately 0.8% per year (approximately 8% in 10 years) [1], and a steep decline in muscle, amounting to approximately 15% in 10 years, is observed in the elderly after 60 years. This change in skeletal muscle mass and muscular strength in relation to aging is defined as sarcopenia [1], which has been reported to be associated with increased functional impairment, fall risk, disability, and mortality in the elderly [2,3,4].

Cardiometabolic diseases, such as metabolic syndrome, atherosclerosis, ischemic heart disease, diabetes, and stroke, are among the leading causes of mortality. Cardiometabolic risk factors, such as abdominal obesity, hypertension, hyperglycemia, dyslipidemia, and hypercholesterolemia, are major risk factors associated with cardiovascular disease and related mortality. Since cardiovascular disease is one of the major causes of death in Korea, its prevention will inevitably emerge as a health promotion strategy.

Many recent epidemiologic studies on sarcopenia have focused on cardiometabolic health and physical dysfunction. Moreover, in their meta-analysis, Chang and Lin reported higher mortality rates in the sarcopenia group than in the non-sarcopenia group [5]. Therefore, preventing sarcopenia may be a key step for preventing cardiometabolic diseases.

The strong evidence from clinical and epidemiological studies suggests that decreased skeletal muscle mass, or sarcopenia, is independently associated with adverse cardiometabolic health and an increased risk of mortality [5,6,7]. The Asian Working Group on Sarcopenia has recommended that sarcopenia should be assessed using both skeletal muscle mass and muscle strength, as well as physical function, such as handgrip strength [8]. Previous studies have reported that decreased muscular strength, such as handgrip strength, may be more associated with increased mortality, late-life disability, hospital admissions, cognitive decline, and poor quality of life in older adults [9,10,11,12,13] than skeletal muscle mass. However, current evidence focuses on skeletal muscle mass, with only a few studies demonstrating the association between muscular strength and cardiometabolic health in the US and Taiwan [14,15]. Whether these cardiometabolic outcomes of handgrip strength may be generalized to the global population remains unclear [16], and has not been evaluated in the Korean population.

Therefore, the objective of the present study was to establish the association between handgrip strength and the risk of adverse cardiometabolic health in a representative adult Korean population. We also investigated the sex and age differences in the associations between handgrip strength and adverse cardiometabolic health, as the decrease in strength with aging may differ between male and female individuals [17].

## 2. Materials and Methods

### 2.1. Study Participants

Data from the Korea National Health and Nutritional Examination Survey (KNHANES) were used for this study. The KNHANES is a series of cross-sectional surveys of nationally representative samples of a civilian, non-institutionalized population, conducted with the purpose of assessing the health and nutritional status of the Korean population. These surveys were approved by the Institutional Review Board of the Korea Centers for Disease Control and Prevention, and all of the participants provided written informed consent.

For this study, we selected 9246 adults aged ≥ 40 years from a total of 16,277 participants. We excluded 804 participants owing to missing data for fasting blood samples, and 485 due to socio-demographic and lifestyle characteristics (education level, household income, smoking status, alcohol consumption, and physical activity). We also excluded 355 participants due to missing data for handgrip strength. Finally, 7602 participants were included in the study population.

### 2.2. Handgrip Strength

In this study, the maximum grip strength was used to evaluate the muscular strength level of the participants. The handgrip strength was measured three times for each of the left and right hands, and the average value was calculated. Relative handgrip strength (%) was calculated by dividing weight by handgrip strength multiplied by 100 [18].

### 2.3. Cardiometabolic Risk Factors

Cardiometabolic risk factors included waist circumference (WC, cm), systolic blood pressure (SBP, mmHg) and diastolic blood pressure (DBP, mmHg), fasting blood glucose (FBG, mg/dL), glycosylated hemoglobin (HbA1c, %), triglycerides (TG, mg/dL), high-density lipoprotein (HDL) cholesterol (mg/dL), and total cholesterol (TC, mg/dL). All cardiometabolic risk factors were defined based on previous studies in the Korean population [19].

Abdominal obesity was defined as WC ≥ 90 cm for men and ≥80 cm for women. High FG was defined as an 8-h fasting glucose level of ≥100 mg/dL. High glycosylated hemoglobin was defined as HbA1c ≥ 6.5%, and high total cholesterol as ≥190.0 mg/dL. Low HDL cholesterol was defined as <40 mg/dL for men and <50 mg/dL for women. High blood pressure was defined as an SBP of ≥130 mmHg and/or DBP of ≥85 mmHg. Finally, we calculated the total number of adverse cardiometabolic risk factors as the sum of each adverse risk factor, and defined high risk as having ≥three adverse cardiometabolic risk factors [20,21].

### 2.4. Covariates

Several variables, such as sociodemographic factors (age, sex, education level, and household income) and health-related behaviors (smoking status, alcohol consumption, moderate-to-vigorous physical activity), and anthropometric measurements (BMI) were used as confounding factors. Educational level was categorized as < high school, high school, and > high school. Household income was categorized into quartiles based on each survey year. Smoking status was categorized into never, former, and current. Alcohol consumption was categorized as never, once a week, two to four times/week, and ≥four times/week. BMI (kg/m^2^) was calculated by body weight (kg) divided by height (m^2^). Moderate-to-vigorous physical activity (MVPA) was categorized into low (< 150 min/week) and high (≥ 150 min/week).

### 2.5. Statistical Analysis

All analyses were performed using the Statistical Analysis System (SAS) survey procedures (ver. 9.4, SAS Institute, Cary, NC, USA) to account for the complex survey design, survey non-response, and post-stratification. The study participants’ characteristics were described using descriptive statistics and calculated weighted percentage (weighted %). Statistical significance was analyzed using the Rao–Scott chi-square tests. We used a survey regression model to estimate the weighted mean and standard error (SE) for continuous variables according to sex and category of handgrip strength quartiles. To assess the effects of potential confounders in the association of handgrip strength level and adverse cardiometabolic health (having ≥three risk factors), we developed three sequential logistic regression models: model 1 was adjusted for demographic factors, including age, sex, education level, and household income; model 2 was additionally adjusted for smoking status, alcohol consumption, and MVPA levels; finally, in the fully adjusted model, we additionally adjusted for the body mass index.

We also calculated the odds ratio (OR) and confidence interval (CI) for adverse cardiometabolic risk according to the category of handgrip strength. In addition, we examined sensitivity analysis using the stratified models by sex (men or women) and age group (< 65 years or ≥ 65 years) to evaluate sex and age-related differences in these associations.

## 3. Results

For this study, a total of 7602 participants (3383 men, 4219 women) aged ≥ 40 years had available data (Table 1). Overall, patients’ mean age was 56.40 ± 0.22 years, and mean handgrip strength was 30.24 ± 0.15 kg. The mean WC, FBG, and HbA1c were 83.60 ± 0.16 cm, 104.01 ± 0.37 mg/dL, and 5.80% ± 0.01%, respectively. Of the mean WC, FBG, HbA1c, systolic and diastolic blood pressure, TG, and total cholesterol were significantly higher in men than in women (Table 1, all *p* < 0.001). However, the HDL and total cholesterol levels were significantly higher in women. The mean total number of cardiometabolic risk factors was 2.78 ± 0.03 in men and 2.45 ± 0.03 in women (*p* < 0.001).

Table 2 presents the participants’ characteristics, individual cardiometabolic risk factors, and adverse cardiometabolic health risk (having ≥three risk factors) according to the category of handgrip strength level. All cardiometabolic risk factors differed significantly according to the category of handgrip strength levels (all *p* < 0.001). The percentage of adverse cardiometabolic health was 69.4% in the lowest quartile of handgrip strength, and 31.1% in highest quartile (Table 2, *p* < 0.001).

Table 3 shows the association between handgrip strength and high risk of adverse cardiometabolic health using different multivariate logistic regression models. Handgrip strength level was significantly associated with an increased risk of adverse cardiometabolic health in all sequential models (all *p*-Values < 0.05). In the fully adjusted model (Model 3), adjusted OR for cardiometabolic health was 0.76 (95% CI: 0.61–0.94) in the highest quartile compared to the lowest quartile (reference).

We conducted a sensitivity analysis using models stratified by sex (men or women) and age group (<65 years or ≥65 years). Using fully adjusted models, the OR for adverse cardiometabolic health in the highest quartile was 0.67 (95% CI: 0.50–0.91, *p*-Value < 0.001) in men and 0.87 (95% CI: 0.66–1.14) in women. Moreover, when we conducted sensitivity analysis using models stratified by age group, significant associations were observed in both middle-aged (OR = 0.76 in highest quartile versus reference group) and older adults (OR = 0.68) (Table 4).

## 4. Discussion

We are the first to describe the associations between categories of handgrip strength and adverse cardiometabolic risk factors among a large representative sample of Korean adults. The key finding was that when compared to lower handgrip strength, higher strength was independently associated with a lower adjusted risk of adverse cardiometabolic health. Our findings for handgrip strength suggest that muscular strength is independently associated with optimal cardiometabolic health among Korean adults.

In the present study, the risk of adverse cardiometabolic health was approximately 24% lower in the participants with higher handgrip strength than in those with lower muscular strength. This result also did not change after adjusting for potential confounding factors. Compared with similar cross-sectional studies, we found comparable beneficial cardiometabolic health results for higher handgrip strength. Furthermore, our findings support the findings from emerging cross-sectional population studies which suggest that compared to lower handgrip strength, higher strength is associated with lower metabolic biomarkers and the metabolic syndrome [18,22]. For instance, a previous cross-sectional survey on 5520 individuals from the general Chinese population reported that decreased handgrip strength was associated with increased derangements of the metabolic profile and metabolic diseases [18]. In addition, Kawamoto et al. (2018) identified that a higher level of handgrip strength was associated with cardiometabolic disorders among community-dwelling Japanese individuals [22]. Other studies have shown that sarcopenia caused by an age-related decrease in skeletal muscle was associated with an increased risk of cardiovascular disease, diabetes, metabolic syndrome, and mortality [23,24,25]. The findings of this study suggest that health promotion strategies can further emphasize the importance of muscle strength for cardiometabolic health in the Korean population.

Skeletal muscle decreases by approximately 0.8% per year, which further declines after the age of 60 years [26]. In general, muscle strength is known to be proportional to the amount of skeletal muscle, and the decrease in skeletal muscle due to aging causes degradation of muscular function. In experimental clinical studies, the skeletal muscle has been found to play an important role in the regulation of glucose metabolism and blood lipids. Moreover, changes in skeletal muscle mass and strength may reflect changes in energy metabolism with aging. In a recent Korean study, handgrip strength was found to be positively associated with the basic metabolic rate (BMR) in 2512 older adults [27]. Thus, a decrease in strength may reflect a loss of skeletal muscle mass.

However, handgrip strength may affect the cardiometabolic health independent of skeletal muscle mass. A recent epidemiological study reported a disassociation between muscle mass and strength [16]. In addition, in a clinical study, taking androgen or growth factor supplementation contributed to a significant improvement in the body composition, including muscle mass, but not muscular function or performance [28]. A prospective cohort study in half a million adults in the UK showed that handgrip strength was associated with cardiovascular disease, cancer, and all-cause mortality [9]. These results may suggest that muscle strength may play a different role in human health, irrespective of muscle mass. Clark and Manini (2008) [16]. introduced the term “dynapenia”, as sarcopenia was limited to the original definition of loss in skeletal muscle mass with aging; this reflects the need to focus on these variables when determining their role in sarcopenia.

Decreased muscle mass and strength may reflect an increase in inflammation in the human body [29]. Previous observational and review studies showed that sarcopenia is associated with elevated levels of inflammatory cytokine IL-6, IL-6/IL-10, and anti-inflammatory cytokine IL-10 [26,29,30]. Moreover, a recent clinical study among hospitalized older patients reported that acute inflammation is associated with decreased muscular strength, mass, and function [31]. In addition, increased chronic inflammation, even at low levels, is associated with adverse metabolic biomarkers, elevated risk of metabolic syndrome, cardiovascular disease, and mortality [29,32,33]. In conjunction, the loss of muscle strength and mass may affect the adverse cardiometabolic health through increased inflammation [26].

In the present study, we also investigated the sex differences in the association between handgrip strength and the risk of adverse cardiometabolic health. In a sex-stratified analysis among men, higher handgrip strength resulted in a significantly lower risk of adverse cardiometabolic health compared to lower strength. In contrast, significantly lower odds ratios were not observed across all three categories of handgrip strength among women. Many previous studies have reported that muscle mass and strength are related to metabolic diseases and biomarkers in both men and women. However, several studies also showed sex differences in the association of muscle mass or strength and cardiometabolic health [18,34,35]. In agreement with our findings, Li et al. reported that higher handgrip strength was associated with a lower risk of impaired fasting glucose level in men alone [35]. Several reasons may explain the sex difference in the associations between handgrip strength and cardiometabolic health. The physiological difference by sex suggests that the level of testosterone is linked to both cardiometabolic risk factors and skeletal muscle mass [36,37,38]. Data from the Study of Women’s Health Across the Nation (SWAN) suggest that menopause-related testosterone is associated with visceral fat accumulation and adverse cardiovascular risk in middle-aged women [39]. Moreover, a clinical intervention study showed that taking testosterone supplementation for three years improved muscle performance and physical function in the elderly population [40]. Estrogen also has a protective effect on inflammation-related loss of skeletal muscle among women [17]. In view of the cross-sectional design, strong inferences from these sex differences should be drawn with caution.

The strength of this study was the use of a representative nationwide sample of Korean adults. Secondly, in our coordinated analysis, we controlled for several socio-demographic factors, such as age, sex, education, and household income, and health-related behaviors, such as smoking, alcohol consumption, and physical activity. Finally, we used standardized data and biological samples from a representative population of Korean adults; this made it comparable to the KNHANES and similar studies. However, our study had several limitations. First, the cross-sectional design may have limited the inference of causality between handgrip strength and adverse cardiometabolic health. Therefore, future longitudinal studies are needed to investigate the direction of causality between categories of handgrip strength and adverse cardiometabolic health. Although handgrip strength reflects the muscular strength of the whole body, there may be limits to its ability as an indicator of muscular strength. Moreover, we used only handgrip strength as the parameter for muscular strength, and did not use skeletal muscle mass. Finally, since KNHANES did not measure hormone-related variables, we could not consider hormone-related variables, such as testosterone, in models. Future research is needed to evaluate the effects of muscular strength and mass on cardiometabolic health, and to determine the effects of long-term interventions.

## 5. Conclusions

In conclusion, we investigated the association between handgrip strength and adverse cardiometabolic health among a representative Korean population using KNHANES data. Higher handgrip strength was significantly associated with adverse cardiometabolic health. Our findings suggest that maintaining higher handgrip strength may contribute to improved cardiometabolic health. However, this relationship is more evident in men than in women.

## Figures and Tables

**Table 1:** Participants’ characteristics overall, in males and in females.

Characteristics	Overall (*n* = 7602)		Male (*n* = 3383)		Female (*n* = 4219)		*p*-Value
Age (ears) ^1^	56.40	±0.22	55.75	±0.24	57.04	±0.25	<0.001
Handgrip strength (kg)	30.24	±0.15	38.51	±0.18	22.19	±0.11	<0.001
Related handgrip strength (%)	46.78	±0.20	55.21	±0.24	38.58	±0.21	<0.001
BMI (kg/m^2^)	24.19	±0.05	24.47	±0.06	23.91	±0.07	<0.001
Waist circumference (cm)	83.60	±0.16	86.70	±0.18	80.59	±0.23	<0.001
Fasting blood glucose (mg/dL)	104.01	±0.37	106.99	±0.53	101.11	±0.45	<0.001
Hemoglobin A1c (%)	5.80	±0.01	5.84	±0.02	5.76	±0.02	<0.001
Systolic blood pressure (mmHg)	120.93	±0.28	122.19	±0.34	119.70	±0.37	<0.001
Diastolic blood pressure (mmHg)	77.02	±0.17	79.13	±0.24	74.96	±0.19	<0.001
HDL cholesterol (mg/dL)	50.19	±0.18	46.73	±0.22	53.56	±0.25	<0.001
Triglyceride (mg/dL)	148.83	±2.16	173.26	±3.94	125.06	±1.65	<0.001
Total cholesterol (mg/dL)	196.64	±0.57	193.79	±0.86	199.41	±0.65	<0.001
Cardiometabolic risk factors (numbers)	2.61	±0.03	2.78	±0.03	2.45	±0.03	<0.001
Education (*n* (%)) ^2^							
<High School	3138	(34.7)	1153	(27.9)	1985	(41.3)	<0.001
High School	2235	(32.0)	1008	(31.1)	1227	(32.8)	
>High School	2229	(33.3)	1222	(41.0)	1007	(25.9)	
Household income (*n* (%))							
Q1	1719	(18.4)	679	(15.3)	1040	(21.5)	<0.001
Q2	1845	(22.7)	825	(22.4)	1020	(23.0)	
Q3	1887	(27.0)	856	(27.5)	1031	(26.4)	
Q4	2151	(31.9)	1023	(34.9)	1128	(29.1)	
Alcohol consumption (*n* (%))							
Never	2426	(28.0)	694	(17.3)	1732	(38.3)	<0.001
Once a week	3511	(47.7)	1401	(43.1)	2110	(52.1)	
Two–three times/week	1064	(16.1)	785	(25.1)	279	(7.3)	
≥Four times/week	601	(8.3)	503	(14.4)	98	(2.3)	
Smoking status (*n* (%))							
Never	4642	(57.1)	715	(20.9)	3927	(92.4)	<0.001
Former	1725	(23.6)	1590	(44.5)	135	(3.2)	
Current	1235	(19.3)	1078	(34.6)	157	(4.4)	
MVPA (*n* (%))							
Low	6523	(83.6)	2782	(79.7)	3741	(87.4)	<0.001
High	1079	(16.4)	601	(20.3)	478	(12.6)	

^1^ Weighted mean ± SE from survey mean. ^2^ Weighted percentages from survey frequency (all such values). Cardiometabolic risk factors were calculated as the sum of each risk factors including abdominal obesity, high fasting glucose, high glycosylated hemoglobin, low HDL cholesterol, high blood pressure. BMI, body mass index; HDL, high-density lipoprotein; MVPA, moderate-to-vigorous physical activity; SE, standard error.

**Table 2:** Characteristics of study population by category of handgrip strength.

Characteristics	Handgrip Strength Level								*p*-Value
	Q1 (*n* = 1894)		Q2 (*n* = 1855)		Q3 (*n* = 1908)		Q4 (*n* = 1945)		
Age (years) ^1^	62.40	±0.40	57.24	±0.32	55.13	±0.28	52.18	±0.24	<0.001
Percentage of men (n (%)) ^2^	808	(45.4)	876	(51.3)	852	(50.8)	847	(49.3)	0.016
Lowest educational level (n (%))	1144	(53.4)	813	(37.8)	673	(29.6)	508	(22.0)	<0.001
Lowest quartile of household income (n (%))	726	(33.4)	419	(18.5)	359	(15.8)	215	(9.1)	<0.001
Highest alcohol consumption (n (%))	126	(6.7)	168	(9.4)	170	(9.3)	137	(7.6)	<0.001
Current smoker (n (%))	254	(16.3)	286	(18.0)	327	(20.6)	368	(21.6)	0.004
Higher MVPA (n (%))	153	(9.3)	248	(15.3)	332	(20.4)	346	(19.2)	<0.001
Individual adverse cardiometabolic risk factors									
Abdominal obesity (n (%))	1063	(56.8)	770	(41.4)	465	(24.4)	181	(9.2)	<0.001
High fasting blood glucose (n (%))	1095	(57.6)	898	(47.6)	800	(41.8)	665	(34.0)	<0.001
High Hemoglobin A1c (n (%))	409	(20.7)	273	(13.5)	185	(9.3)	123	(6.1)	<0.001
High blood pressure (n (%))	1228	(63.0)	1043	(52.5)	925	(46.5)	740	(37.3)	<0.001
Low HDL (n (%))	928	(48.4)	694	(35.6)	637	(31.9)	512	(25.4)	<0.001
High TG (n (%))	717	(40.1)	671	(38.3)	591	(32.3)	489	(25.6)	<0.001
High total cholesterol (n (%))	906	(51.9)	1010	(57.4)	1091	(58.1)	1131	(58.1)	<0.001
Adverse cardiometabolic risk factors, ≥3 (n (%))	1310	(69.4)	1043	(55.3)	878	(45.9)	621	(31.1)	<0.001

^1^ Weighted mean ± SE from survey mean. ^2^ Weighted percentages from survey frequency (all such values). Adverse cardiometabolic health defined as having ≥3 risk factors. MVPA, moderate-to-vigorous physical activity; HDL, high-density lipoprotein; TG, Triglyceride; SE, standard error.

**Table 3:** Odds ratio (OR) (95% CIs (confidence intervals)) for adverse cardiometabolic health by category of handgrip strength.

Handgrip Strength	Model 1		Model 2		Model 3	
	OR	(95% CI)	OR	(95% CI)	OR	(95% CI)
Q1 (*n* = 1894)	1.00	(reference)	1.00	(reference)	1.00	(reference)
Q2 (*n* = 1855)	0.78	(0.65–0.92) *	0.81	(0.68–0.96) *	0.81	(0.68–0.96) *
Q3 (*n* = 1908)	0.77	(0.64–0.93) *	0.80	(0.67–0.97) *	0.81	(0.67–0.98) *
Q4 (*n* = 1945)	0.71	(0.58–0.87) *	0.75	(0.61–0.92) *	0.76	(0.61–0.94) *
*p*-Value	0.003		0.015		0.023	

Model 1: adjusted for age, sex, education level, household income. Model 2: adjusted for Model 1 covariates plus smoking status, alcohol consumption, and physical activity level. Model 3: adjusted for Model 2 covariates plus body mass index. * *p*-value < 0.05 versus reference group.

**Table 4:** OR (95% CIs) for adverse cardiometabolic health by category of handgrip strength by sex and age group.

Handgrip Strength	No.	OR	(95% CI)	No.	OR	(95% CI)
		Men (*n* = 3383)			Women (*n* = 4219)	
Q1	808	1.00	(reference)	1086	1.00	(reference)
Q2	876	0.87	(0.69–1.10)	979	0.89	(0.75–1.08)
Q3	852	0.69	(0.52–0.91) *	1056	0.96	(0.78–1.22)
Q4	847	0.67	(0.50–0.91) *	1098	0.87	(0.66–1.14)
*p*-Value		<0.001			0.736	
		Age < 65 years (*n* = 5033)			Age ≥ 65 years (*n* = 2569)	
Q1	794	1.00	(reference)	1100	1.00	(reference)
Q2	1156	0.79	(0.63–1.00)	699	0.84	(0.65–1.08)
Q3	1415	0.81	(0.63–1.03)	493	0.67	(0.50–0.89) *
Q4	1668	0.76	(0.58–0.99) *	277	0.68	(0.47–0.98) *
*p*-Value		0.117			0.005	

Models were used fully adjusted model used in Table 3 (Model 3). * *p*-Value < 0.05 versus reference group.
